# Evaluation of the Viability of 3D Printing in Recycling Polymers

**DOI:** 10.3390/polym16081104

**Published:** 2024-04-16

**Authors:** Chrysanthos Maraveas, Ioannis Vasileios Kyrtopoulos, Konstantinos G. Arvanitis

**Affiliations:** Department of Natural Resources Development and Agricultural Engineering, Agricultural University of Athens, 75 Iera Odos Street, 11855 Athens, Greece; kyrtopoulos@aua.gr (I.V.K.); karvan@aua.gr (K.G.A.)

**Keywords:** 3D printing, polymers, recycling, photolysis, degradation, waste, filaments

## Abstract

The increased use of plastics in industrial and agricultural applications has led to high levels of pollution worldwide and is a significant challenge. To address this plastic pollution, conventional methods such as landfills and incineration are used, leading to further challenges such as the generation of greenhouse gas emissions. Therefore, increasing interest has been directed to identifying alternative methods to dispose of plastic waste from agriculture. The novelty of the current research arose from the lack of critical reviews on how 3-Dimensional (3D) printing was adopted for recycling plastics, its application in the production of agricultural plastics, and its specific benefits, disadvantages, and limitations in recycling plastics. The review paper offers novel insights regarding the application of 3D printing methods including Fused Particle Fabrication (FPF), Hot Melt Extrusion (HME), and Fused Deposition Modelling (FDM) to make filaments from plastics. However, the methods were adopted in local recycling setups where only small quantities of the raw materials were considered. Data was collected using a systematic review involving 39 studies. Findings showed that the application of the 3D printing methods led to the generation of agricultural plastics such as Polylactic Acid (PLA), Acrylonitrile Butadiene Styrene (ABS), Polyethylene Terephthalate (PET), and High-Density Polyethylene (HDPE), which were found to have properties comparable to those of virgin plastic, suggesting the viability of 3D printing in managing plastic pollution. However, limitations were also associated with the 3D printing methods; 3D-printed plastics deteriorated rapidly under Ultraviolet (UV) light and are non-biodegradable, posing further risks of plastic pollution. However, UV stabilization helps reduce plastic deterioration, thus increasing longevity and reducing disposal. Future directions emphasize identifying methods to reduce the deterioration of 3D-printed agricultural plastics and increasing their longevity in addition to UV stability.

## 1. Introduction

The use of plastics and polymers has significantly increased in society. Hunt et al. [1] and Pinho, Amaro, and Piedade [2] report that the use of plastics in contemporary society has increased due to technological development and a rise in population. Over the past years, production of plastics has risen by about 500% [2]. Karimi [3] also posits that most plastics derived from petroleum cannot be degraded, which leads to increased oil consumption and environmental pollution. Furthermore, the methods employed to dispose the plastics are unsustainable and generate adverse environmental effects. The authors of [1] observe that conventional methods of disposing of plastics, such as burying them in soil, are unreliable. Devasahayam [4] adds that burnt plastics and polymers produce high amounts of carbon dioxide, which accumulates in the upper atmosphere. As a result, the unreliable incineration disposal methods for non-biodegradable plastics lead to global warming and climate change effects [5,6]. A further consequence of the impact of waste plastics that escape into the environment are microplastics [7]. Allen et al. [8] add to [6], highlighting the diverse repositories of microplastics, including sea water, which releases them due to the action of bubble burst ejection and wave action. Tong et al. [9] also observe that micro- and nano plastics can be formed during the degrading of biodegradable plastics (such as polystyrene, polyvinyl chloride, and polylactic acid among others) and their exposure to continuous UV. In other studies, [10] linked the increased release of microplastics to the use of surgical masks during the COVID-19 pandemic and wet wipes. From the evaluation of [8,9], microplastics and nano plastics are shown to escape into the air due to poor disposal strategies. Disposal of surgical masks to the land surface and the degradation of the plastics due to exposure to UV propagates the nano plastics into the atmosphere.

An alternative approach to disposing plastics is the use of recycling. Pinho, Amaro, and Piedade [2] and Madhu et al. [11] recommend recycling as a critical method for reducing the amount of plastic and polymer waste disposed in landfills and the use of more raw materials to produce more plastic and polymer products. Likewise, Voet, Guit, and Loos [12] note that using post-consumer polymer materials in production is a reliable way of addressing the plastic menace without producing greenhouse gases. Tsuchimoto and Kajikawa [13] identify four types of recycling adopted for plastics; primary (re-extrusion), secondary (mechanical), tertiary (chemical), and quaternary (energy recovery). With the primary recycling method, plastics are converted into products that have similar performance characteristics as virgin plastics, for example, generating new PET bottles from postconsumer bottles [13,14]. Klotz, Haupt, and Hellweg [15] support [13] and reveal that in secondary recycling, the generated products from the recovered plastics have less performance characteristics than the virgin plastics such as tiles made from mixed polyolefins. The chemical recycling method encompasses methods such as pyrolysis, gasification, and solvolysis where the virgin plastics are converted into their original monomers or chemicals used in production of high-quality plastics [16]. The final method, energy recovery, is not ranked as recycling method since it involves the extraction of energy in form of heat from the virgin plastics [12]. The literature review reveals that the use of the various recycling methods for plastic disposal has been widely examined. Current findings indicate that different types of recycling are reliable in reducing greenhouse gas emissions following the incineration of polymers.

Despite the evidence of recycling as a reliable method of disposing non-biodegradable plastics, there is a limited understanding of employing advanced methods of recycling plastics, particularly the use of 3D printing. The novelty of the current research is its emphasis on the use of 3D printing as a method for recycling plastics. According to Mikula et al. [17], 3D printing has emerged as a reliable method of recycling plastics. Similarly, Hunt et al. [1] support [17], arguing that plastic wastes can be used in making filaments for 3D printing. The studies indicate that waste plastics are crushed into flakes and hot extruded to make 3D printing filaments that have similar chemistry as virgin polymers. However, Chong et al. [18] reported that while the public has demonstrated an exponential increase in understanding the importance of recycling polymers, there is a generally limited awareness about recycling methods, for instance, additive manufacturing methods like 3-D printing. The authors of [1] note that additive manufacturing reduces plastic pollution by reducing the waste generated from plastics while putting them under meaningful use. For instance, polymer-based filaments for 3D printing are produced from used plastics rather than synthesized from petrochemicals and radiations to make polymers with chemistry similar to existing plastic waste. In 3D printing, complex polymer products are produced by modeling recycled plastics with the help of a Computer-Aided Design (CAD) model. 

According to Kazmer [19], the process of 3D printing entails depositing, joining, and solidifying a combination of materials, for instance, plastics, powder grains, and petrochemicals, under the control of a computer to create a 3-dimensional product of a predetermined shape. Open-source 3D printers have increased the use of recycled polymers and plastics in making domestic and fashion items such as jewelry and have rapidly prototyped new ideas [20]. All the benefits prove critical in reducing environmental pollution compared to conventional manufacturing and recycling techniques. As a result, they have become an economically viable investment among the average US household. Their adoption in recycling will likely be beneficial in managing plastic and polymer wastes since more waste will be recycled rather than landfilled. Oussai, Bártfai, and Kátai [21] also found that 3D printing is prominent in recycling polymers because it is cheap yet reliable for producing functional components. The technique is lauded as a clean, sustainable processing technology, since it facilitates the transformation of consumer polymer and plastic waste into new components [22]. Karimi et al. [23] further report that 3D printing techniques such as Fused Deposit Modeling (FDM) are popular due to their ease of use, low cost, high efficiency, and safety. Therefore, [22,23] emphasize that 3D printing supports circular economic goals given that it helps address plastic contamination and limit over-reliance on methods, such as incineration, that account for the highest amount of carbon dioxide emissions that have accelerated global warming, promoting climate change.

Local recycling processes describe the small-scale activities employed to recycle plastics using 3D printing techniques. Embracing local recycling processes in 3D printing contributes to the circular economy as plastics that have reached their end of life are transformed to new uses. Despeisse et al. [24] posit that the circular economy aims to enhance the efficiency of resources in society by eliminating waste, hence causing a shift away from the conventional linear model that leads to more waste. Chin [25] supports [24] and explains that in the circular economy, the use of recycled resources reduces the demand on the extraction of new resources while preventing impact along the processing chain. As such, the comparison of studies [24,25] indicates that the circular economy is integral in reducing waste by transforming it into new uses. Further study examines the influence of 3D printing processes in the circular economy. In one study, Al Rashid and Koç [26] reveal that integrating the circular economy in 3D printing processes can contribute to a synergic impact and generating new uses of disposed polymers and plastics. The authors of [24] also reveal that 3D printing adds to the circular economy by ensuring that waste materials are reused directly as input, hence leading to a closed-loop circulation of materials. Santander et al. [27] add to [24,26] and observe that 3D printing contributes to closed-loop supply chains as inventory is reduced, disassembling efforts are minimized, on-demand spare parts are increased, and personalization is further enhanced. Valera et al. [28] further observe that the use of 3D printing supports the reduction of waste while increasing the lifetime of materials, hence contributing to the circular economy by creating customized local solutions in multiple industries. The insights from the comparison of [24,26,27,28] indicates that the use of 3D printing polymers helps reuse harmful plastic waste both in personal and industrial uses at a lower cost. 

**Figure 1:**
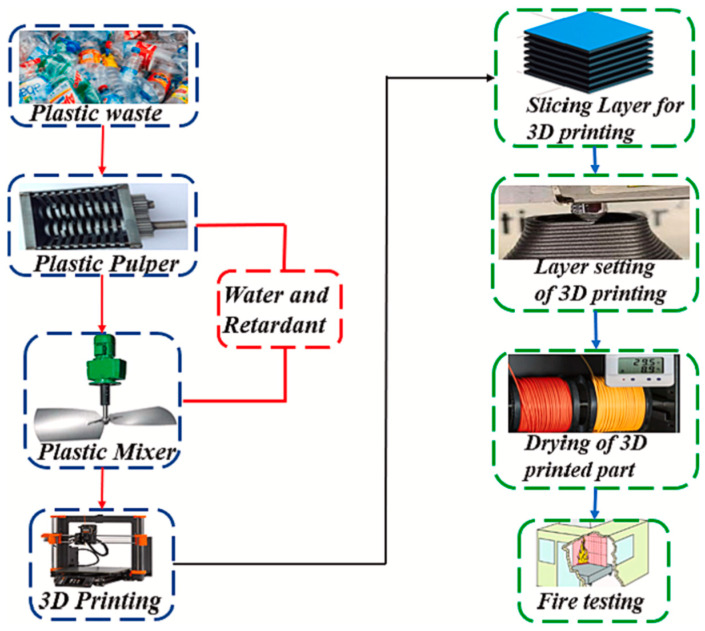
Using 3D printing to recycle plastic waste [29].

Further advantages emerge in the use of 3D printing recycling processes to contribute to innovative designs based on the ease of customization. Olawumi et al. [29] explain that 3D printing techniques are integral in creating objects that are easily customized, for instance, printable filaments. This implies that 3D printing for the recycling of polymers provided unparalleled design freedom and enhances design innovation by enabling the creation of unique products that meet specific preferences. A similar observation is made in the construction industry, where the use of geopolymer-based 3D printed construction materials generated the least global warming potential when compared to structures based on Portland cement [30]. The inference from [28,29] is that 3D printing processes used in recycling polymers and plastics are beneficial in developing new customized products that enhance design, lower consumption of energy, and minimize the environmental impact of the materials. Peeters, Kiratli, and Semeijn [31] also add that 3D printing in recycling democratizes manufacturing processes and leads to the recyclability of new products, for example, thermoplastics. As such, local recycling processes using 3D printing are pivotal in promoting environmental sustainability and enhancing innovation processes by contributing to new materials. Figure 1 illustrates the use of 3D printing to recycle plastic waste.

In Figure 1, the use of 3D printing to recycle plastic waste is identified where the process involves the use of plastic pulpers and mixers. The outcome of the recycled plastics is materials used as feedstock in 3D printing processes. As such, 3D printing is a strategy to facilitate the recycling of plastic wastes.

In other studies, different products are identified to emerge from 3D printing processes. For example, Oyinlola et al. [32] reveal that the use of 3D printing to convert plastic waste to filaments has a significant implication on the local economy by allowing plastic waste to be converted into more valuable products. Hassan, Mohanty, and Misra [33] also report that 3D printing is used in the upcycling of waste biomass generated from agriculture; this contributes to sustainable processes, reduces the overall waste generated, and enhances design and production processes. Patel et al. [34] support Mohanty and Misra [33] and add that 3D printing is useful in the manufacture of carbon fiber-reinforced polymers that contain recycled plastic wastes. Bayati et al. [35] also demonstrated that 3D-printed elastomer from propylene waste material exhibited an elongation exceeding 4000% and hence the material was suitable in soft robotics and flexible electronics applications. The insights underscore the broad application areas of 3D printing in recycling plastics. 

The use of 3D printing in recycling plastics in the real-world scenario, including mixed plastic streams and different grades of the same materials, has been examined in different studies. In one study, Zander et al. [36] argued that most plastic waste comprised different mixtures of polymers, for example, caps and water bottles that comprised polyethylene terephthalate (PET) and polypropylene (PP). The mixed polymers were used as feedstocks in fused filament fabrication (FFF); they were not separated and blended into filaments. The authors of [36] reported that blends of PP/PET compatibilized with styrene ethylene butylene styrene (SEBS) demonstrated high tensile strength (23 MPa) while the PP/PS blends reduced tensile strength to 19 MPa. As such, mixed plastic streams could be used as feedstocks for the manufacture of filaments using 3D printing techniques. Cafiero et al. [37] also demonstrated that plastic waste with different grades of the same materials, such as waste of electrical and electronic equipment (WEEE), could be recycled using 3D printing techniques to manufacture filaments. The study revealed that 11 different polymer blends were associated with the WEEE plastics, which were styrene-based. Despite the different grades of the same polymers, the WEEE plastics were used as feedstocks, during which time they were cleaned, reduced to less than 4mm, and extruded in filaments with the appropriate diameter.

The present paper aims to explore and review the literature to provide evidence of how 3D printing is used in recycling and its application in the production of agricultural plastics. The key research aim was fulfilled by meeting the following objectives:

- To find, in detail, the methods and parameters and other aspects (types of polymer materials) used for recycling through 3D printing.
- To investigate the benefits and limitations of using 3D printing for recycling/re-production.
- To identify future directions regarding using 3D printing for recycling/re-production.
- To investigate beneficial applications, especially in low recycling categories like agricultural plastics.

The research aims and objectives were met by answering the question: how and when can 3Dprinting be used in recycling and production? The study will focus on the ways 3D printing is employed in recycling plastic wastes and the specific functional components produced by 3D printing, for instance, for applications in agriculture. The focus also helped demonstrate the process of recycling plastics using the 3D printing technique and evaluate its feasibility as a sustainable plastic waste management technique.

## 2. Risks of Recycling Polymers Using 3D Printing

An assessment of the lifecycle of 3D printed polymers demonstrates a likelihood of 3D printed polymer waste becoming an environmental issue in the future. Pinho, Amaro, and Piedade [2] noted that 3D printing can create more unrecycled waste and unused plastics than the conventional polymer industry. Furthermore, their properties deteriorate over time, suggesting that they can often become functionally obsolete [38]. The inspection of [2,38] reveals that the shortcoming of 3D-printed plastics arises from the deterioration of their properties over time. As a result, they are unsuitable for many applications where long-term use of the plastics is anticipated. Likewise, Zhu et al. [39] found that repeated recycling of plastics for 3D printing filaments is also limited by the deterioration of the valuable properties of the materials with each subsequent recycling cycle, suggesting that 3D printed plastics are likely to be disposed of in landfills or incinerated and produce carbon dioxide. Therefore, the materials cannot be recycled over their entire lifespan, indicating that they must be disposed of at some point in their lifecycle. 

Semi-crystalline PLAs are vulnerable to hydrolytic and thermo-mechanical degradation during melting. Intrinsic viscosity and molecular weight can also decline due to scissions resulting from the polymer being subjected to shear stress and high temperatures [40]. The argument by Atakok, Kam, and Koc [41] indicates that with each recycling cycle, the viscosity of the recycled materials declined, thereby affecting its strength. Similarly, the polymer might undergo hydrolytic, thermal, and photochemical degradation during its use, decreasing its molecular weight. The authors of [2] and Hidalgo-Carvajal et al. [42] also reported deterioration in polymer mechanical and thermal properties when reprocessed. For instance, Di and Yang [43] conducted up to six consecutive injection and extrusion molding cycles, concluding that Young’s modulus, strain and stress at breakpoint, rheological properties, and hardness all decreased. All the changes were linked to scission, which reduces the overall molecular weight of the polymer.

On the contrary, Beltrán et al. [44] found no significant effects of reprocessing on polymer strength and glass transition point (*T_g_*). However, the Cold Crystallization Temperature (*T_cc_*), according to [41], decreased by about 5 °C. The authors of [44] reiterate [41] and link the changes to the polymeric chains’ high mobility and reduction of molecular weight during melting. Another study by Anderson [45] supported [44] and demonstrated that the tensile strength, hardness, and tensile modulus decreased, whereas shear yield strength decreased for 3D-printed recycled PLA filaments. 

However, contrary findings have been reported for ABS. ABS is an amorphous copolymer commonly used in consumer electronic casings, motorcycles, and automobiles. Its excellent mechanical properties, such as rigidity, impact strength, toughness, and strength, have contributed to its prominent use in industrial polymeric materials [46,47]. It is among the most consumed materials worldwide. However, unlike PLA, processing ABS up to five 3D printer extrusion cycles does not alter its mechanical properties. However, variations emerge based on the type of ABS, the specific printing parameters, and the kind of printing cycles accessed. For instance, repeated cycles of reprocessing ABS in a twin-screw extruder did not change its mechanical aspects and main material properties [48]. As a result, the 3D printing recycling method might be limited to specific materials, whose properties are not likely distorted by the extrusion or 3D printing process. For example, [41] noted that neither ABS’s rheological nor thermal properties were distorted following ten reprocessing cycles. A further limitation to recycling plastics using the 3D printing technique concerns the limited resin identification codes in countries like the US. Until today, there are only seven coded plastics for recycling: High-Density Polyethylene (HDPE), Low-Density Polyethylene (LDPE), Polystyrene (PS), Polyethylene Terephthalate (PET), Polyvinyl Chloride (PVC), Polycarbonate (PC), and Other, which commonly refers to mixed plastics, PC, and ABS [19]. This recycling system is hugely limiting. For instance, the Chinese polymer identification and coding system comprises seven diverse classifications, five symbols referring to post-consumer paths, and 140 identification codes [49]. When put in the context of distributed polymer 3D printing, the US system is lacking in producing plastic products, given that some plastics covered in China’s system are not considered in the US polymer coding system [50]. Furthermore, limited evidence about reprocessing 3D printed products indicates that 3D printed wastes might still pose a significant challenge to managing plastic wastes [24]. As 3D printing increases in prominence, its outcomes might become a significant environmental burden. 

## 3. Systematic Review and Analysis

This section focuses on synthesizing evidence about the use of 3D printing in recycling polymers and their respective applications, for instance in agriculture. The section covers the methods of recycling polymers to make 3D printing feedstock, the various polymers recycled to make 3D printing filaments and their properties, the process of recycling polymer wastes, the challenges as well as the limitations to recycling polymer wastes to produce feedstock for 3D printing, and the shortcomings of 3D printed recycled polymer materials.

### 3.1. Literature Search Process

**Figure 2:**
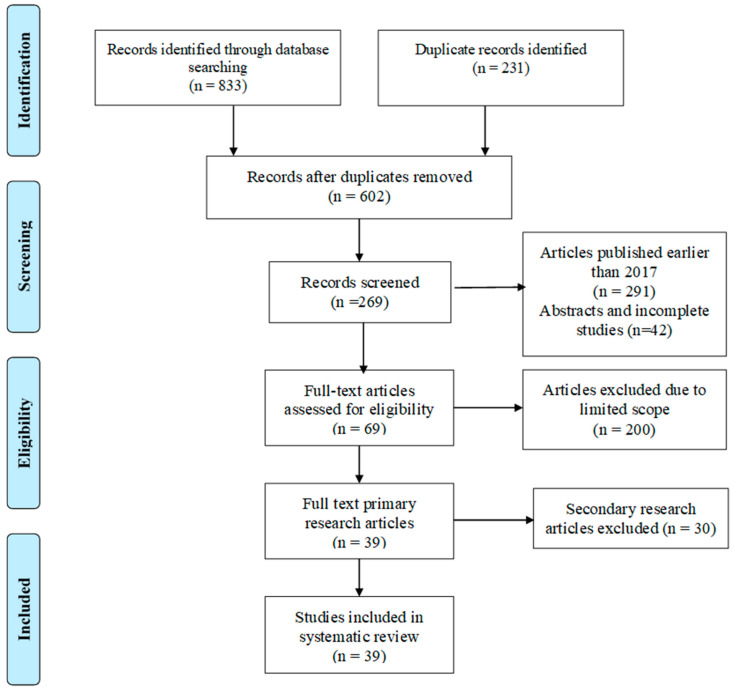
PRISMA Flowchart.

A search was carried out in Scopus, Engineering Village, Springer Materials, JSTOR, Research Gate, and Elsevier. An initial search using the keywords and synonyms led to identification of 833 articles. The articles were sorted based on the inclusion and exclusion criteria to identify the most appropriate articles for synthesizing findings. In the first phase of sorting and filtering, 231 duplicate papers were excluded. The remaining 602 articles were sorted according to the year of publication. Consideration of articles published between 2017 and 2023 led to the exclusion of 291 articles, with 311 articles remaining for further filtering. The papers were sorted according to the scope of the study, focusing on the research topic and 3D printing as a method for recycling polymers. A total of 144 articles were excluded as they focused on issues other than 3D printing as a method for recycling plastics. An additional 56 articles were excluded for solely focusing on a combination of 3D and 4D printing methods for producing construction materials. Subsequently, 111 articles remained for further filtering. Forty-two more articles were eliminated for being abstracts, individual reviews, and incomplete research articles. Subsequent filtering for primary studies on 3D printing as a recycling method led to the exclusion of thirty more studies. Accordingly, 39 research articles remained for review and synthesis of evidence on using 3D printing for recycling plastics. The filtering process is summarized in the Preferred Reporting Items for Systematic Reviews and Meta-Analyses (PRISMA) flowchart shown in Figure 2. Keywords used in the selection of the studies included “3D printing”, “recycling”, “agricultural wastes”, and “polymers”.

In Figure 2, the screening process adopted during the selection of the studies is showcased. Duplicates were removed and the eligible articles further screened against the inclusion and Exclusion criteria. Thirty-nine selected studies were finally considered in the systematic review.

### 3.2. An Overview of the Research Methods Used in the 39 Studies

Two research designs and two research methods were prominent among the thirty-nine studies. Of the 39 studies, 1 was based on a case study design, while 38 were based on the experimental research design. On the other hand, 6 of the 39 studies were based on the mixed-methods research method, while 33 involved a quantitative research methodology. Table 1 showcases the research methods used in the reviewed studies.

In Table 1, the methods used in the reviewed studies were showcased; most of the studies followed a quantitative method. 

According to Thomas [51], a case study involves an in-depth exploration of an activity, program, process, or combination. A specific activity and time bind the target case study. The method is hailed for its high specificity and collection of detailed evidence from the target research subject. Similarly, Heale and Twycross [52] support [51] and argue that evidence obtained from a case study is often more contextual and has a greater depth of specificity. Another advantage of the method is that it can be used in studies involving a small sample, allowing for the generation of in-depth insights into a research problem. However, Sjoberg, Orum, and Feagin [53] contradict [51,52] and argue that case study evidence cannot be generalized to a broader population. The case study method Oussai, Bártfai, and Kátai [21] used in their exploration of the use of recycled Polyethylene Terephthalate (PET) filaments in 3D printing proved critical in demonstrating that 3D printing can support recycling of plastics. The case example also offered in-depth evidence about the properties of recycled PET and their implications on the use of PET in 3D printing applications. The case study design was appropriate given that it aligned with the core aim of the previous study by [21] and the present research problem. 

The experimental research design, on the other hand, entails designing a task to help describe and explain the variation in behavior of a human or non-human subject under different hypothesized conditions [54]. Often, an experiment offers in-depth insights into the cause-effect relationship between variables [55]. Accordingly, explaining the relationship between variables under research, in this case the recycling of polymers and their suitability for 3D printing applications, is critical. The design is also helpful in studying more specific variables through manipulation of observation of the effect [56]. This aspect makes it easy to determine and offer an understanding of the effects of different factors on the subject under study toward affirming groundbreaking hypotheses. The nature of experimental design also gives a researcher more control over the variables they seek to test and the effect [55]. In general, it is a more practical way of establishing the credibility of a relationship between variables under actual rather than simulated contexts. Therefore, it yields the most desirable outcomes, for instance ascertaining the process of recycling polymers and how it affects their properties. In the 38 studies in which the experimental research design was used, the recycling of polymers using different methods and diverse temperature conditions and recycling cycles were actualized, and the respective behaviors of the materials were reported. The experiments led to the identification of the effects of repeated recycling, low and high-temperature applications, exposure to Ultraviolet light (UV), and loading on the properties of 3D-printed recycled polymers. Accordingly, the experimental research design suited the research context by focusing critically on more desirable aspects of recycled polymer filaments and their suitability for various applications.

Quantitative research involves collecting and analyzing numerical data, while mixed research methods entail collecting and analyzing qualitative and quantitative data [57,58]. Quantitative research methods emphasize objective measurement and provide more specific data for ascertaining a cause-effect relationship between variables under study [59]. The measurement aspect was particularly useful in demonstrating the changes in variables, mainly the mechanical properties of polymers following recycling and consistent use. Furthermore, quantitative research, unlike qualitative research, is not based on the researcher’s judgment, but rather on the actual outcomes of measured variables [57]. Therefore, it allows for collecting more authentic evidence, such as the variation in measured properties of recycled and virgin polymers used in 3D printing. 

On the other hand, the mixed methods offset the shortcomings of using either the qualitative or the quantitative research methods [60]. Combining the two methods leads to the synthesis of extensive evidence with in-depth rigor [58,61]. For example, mixed-methods research allows for measurement and observation, crucial in drawing meaningful insights from research data. However, the observation perspective renders the research outcomes more subjective to the researcher’s judgment. Nonetheless, basing the observations on measurements and actual behaviors that manifest during experimentation or measurement guarantees the synthesis of authentic evidence-based findings [61]. Overall, the quantitative and mixed methods methodologies were critical in synthesizing findings that pointed to how 3D printing is used in recycling plastics, the properties of recycled polymers, and the behavior of the recycled 3D printing polymer filaments under different temperatures, lighting, and chemical conditions.

There was a comparative aspect in the 39 studies. Significant comparisons concerned the variation in properties between virgin and recycled polymer feedstock for 3D printing. Esser and Vliegenthart [62] argue that comparative studies provide room for validation of evidence between an experimental and control subject, in this case recycled polymers and virgin polymers. For example, Bergaliyeva et al. [63] and Kumar et al. [64] compared the properties of recycled Polylactic Acid (PLA) and virgin PLA 3D printing filaments to establish the feasibility of 3D printing as a method for recycling plastics. Mohammed et al. [65] compared the properties of recycled ABS and HDPE as standalone polymers and fused in a ratio of 90% ABS and 10% HDPE. The comparisons proved reliable in demonstrating the comparative strength of virgin and recycled polymer filaments for 3D printing. Thus, the comparison aspect in the studies was influential in validating the evidence supporting the feasibility of 3D printing in recycling plastics.

### 3.3. Methods of Recycling Polymers to Make 3D Printing Materials

**Figure 3:**
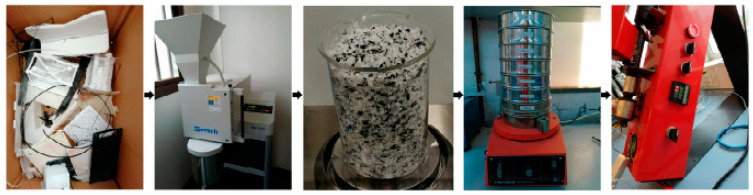
Steps in the Hot Melt Extrusion Process [64].

The methods of recycling polymers identified include Fused Particle Fabrication (FPF), Hot Melt Extrusion (HME), and Fused Deposition Modelling (FDM). FDM as a polymer recycling method was documented in four studies. Hot Melt Extrusion was reported in eight studies: Chu et al. [66], Ji and Jung [67], Atsani and Mastrisiswadi [68], Zhong and Pearce [69], Vones et al. [70], Cisneros-López et al. [71], Beltrán et al. [44], and Zhang, Chen, and Yang [72]. According to Chu et al. [66] and Cisneros-López et al. [71], hot extrusion involves heating and applying pressure to crushed polymer flakes from waste plastics to melt them and force them via an orifice continuously. The method produces thin polymer filaments [44]. The process of preparing materials for hot melt extrusion and the actual extrusion process are summarized in Figure 3.

In Figure 3, the various steps followed in the process of hot melt extrusion are showcased. The polymer was crushed and melted in an orifice. Thereafter, filaments were generated.

FDM involves melting a polymer and dropping it in a modeling orifice or mold [73]. A drop of the molten polymer is consistently added to the FDM machine until the desired material shape and size are achieved [74]. Despite the differences in recycling the polymers, FDM and HME involve crushing the polymers into flakes with subsequent melting. As a result, molten polymers are the primary input at every recycling stage.

### 3.4. Recycling Plastics for 3D Printing

Recycled plastics are mainly used as raw materials for 3-D printing. Sun et al. [75] and Woern et al. [76] assert that 3D printing has resulted in a closed-loop supply chain. Waste materials are used as feedstock for 3D printing processes to produce functional components [75]. The approach renders 3D printing a reliable method for recycling plastic polymers and turning them into meaningful parts that can still be recycled. Santander et al. [27] found that plastic wastes are processed by extrusion to make plastic filaments used as feedstock for 3-D printing or raw materials. Likewise, Gaikwad et al. [77] supported [76] and argued that hot melt extrusion produces 3D printing filaments with different properties that depend on the melting temperature, extrusion pressure, and quantity of added virgin material. The melt extrusion method has mainly increased the use of recycled plastics in 3D printing [36]. Similarly, Woern et al. [76] substantiate that the reliability of plastic recycling methods, such as Fused Particle Fabrication (FPF) and Hot Melt Extrusion (HME), has increased the use of recycled filaments in 3D printing. Arguably, using recycled polymers has become particularly common due to the availability of 3D printing filaments from recycled plastics. Oussai, Bártfai, and Kátai [21] add that Fused Deposition Modelling (FDM) is used in the continuous layering of plastic wastes to create filaments with outstanding mechanical properties for 3D printing applications. Similarly, Gaikwad et al. [77] noted that electronic waste plastics, mainly polycarbonates, are transformed into 3D printing filaments that are then used as raw materials for 3D printing. According to the findings, 3D printing has promoted plastic waste recycling into valuable raw materials for the production of 3D-printed products. Beltrán et al. [44] substantiate that recycled PLA is turned into 3D-printed filaments through melt extrusion with compression molding to industrial standards. Zander et al. [36] found that consumer-grade plastics and polymers are recycled into plastic filaments that are considered a sustainable feedstock for 3D printing. Since separating e-waste is often challenging, blended 3D polymer filaments are produced by the fusion of PET, PLA, and ABS, allowing for the recycling of large amounts of plastic waste [36]. In essence, 3D printing has promoted the recycling of plastic wastes into meaningful raw materials or feedstock. Notably, Pricop et al. [78] established that Polyethylene Terephthalate (PET), the primary material for packing products, is recycled by melting extrusion to make filaments for 3D printing. The material is also used for creating 3D printing prototypes. The findings align with the results from Xu et al. [38] that 3D printing has potentially reduced plastic waste in the surroundings and limited over-reliance on incineration as the only reliable plastic waste management technique. For instance, 3D printing ensures that plastics are reprocessed into functional products, averting risks of carbon dioxide generation following the incineration of the plastic wastes. As a result, the method is more likely to promote the circular economy because it allows for the creation of new 3D-printed products from plastic wastes that could have otherwise been dumped in landfills or incinerated to produce greenhouse gases, including carbon dioxide. The increased use of plastic waste in 3D printing is evidenced by the fact that 3D printing supports rapid prototyping, increasing the production of 3D-printed plastics [79]. Further evidence of the reliability of 3D printing is demonstrated by Pricop et al. [78], who established that 3D printed PET can be recycled up to three times, an indication that 3D printed PET wastes are less likely to accumulate in the surrounding or cause significant alarm because they can have a longer lifespan given the likelihood of recycling them multiple times. Accordingly, 3D printing can be considered a reliable method for recycling plastics and reducing their negative environmental impacts.

### 3.5. Recycled 3D Printing Materials and Their Properties

The primary materials used in 3D printing are presented in the literature matrix in Appendix A. From the table, common materials recycled to make 3D printing feedstock include ABS, PLA, Polyethylene Terephthalate (PET), Nylon, Polypropylene, PVC, HDPE, and LLDPE. However, most studies focused on PET, PLA, and ABS, emphasizing that they are the most common materials used in 3D printing applications. According to Zander et al. [36], a combination of PET, PLA, and ABS wastes results in 3D printing filaments with outstanding mechanical properties. The authors of [36] found that fused PLA, ABS, and PET filaments exhibit exemplary mechanical properties, including a higher tensile strength of up to 35 Mpa. The resulting filaments also have a high glass transition temperature, suggesting they are suitable for high-temperature applications. Likewise, Chu et al. [66] also found that mixing polypropylene and polystyrene wastes by Fuse Filament Fabrication yields 3D printing filaments with 32 MPa more mechanical strength in comparison to their respective virgin materials at extrusion temperatures of about 230 °C. Mohammed et al. [65] also established that a combination of 90% ABS and 10% HDPE produces filaments with consistent 3D prints and mechanical strengths up to 20% higher than the parent virgin material for the respective polymers. The findings consistently demonstrate that combining recycled polymers can produce 3D printing filaments with exemplary mechanical properties. Nonetheless, according to Mendenhall and Eslami [73], repeated heating reduces the radius of curvature of the material. Per Figure 3, [73] demonstrated that the radius of curvature decreases as the material is heated repeatedly at gradually increasing temperatures. ABS and PLA exhibit the properties at temperatures of about 125 °C [73]. Therefore, while the 3D printed recycled polymers exhibit strength comparable to that of virgin filaments, it cannot be curved at a larger radius because it is likely to fail or break. As a result, the application of the recycled filaments might be limited.

**Figure 4:**
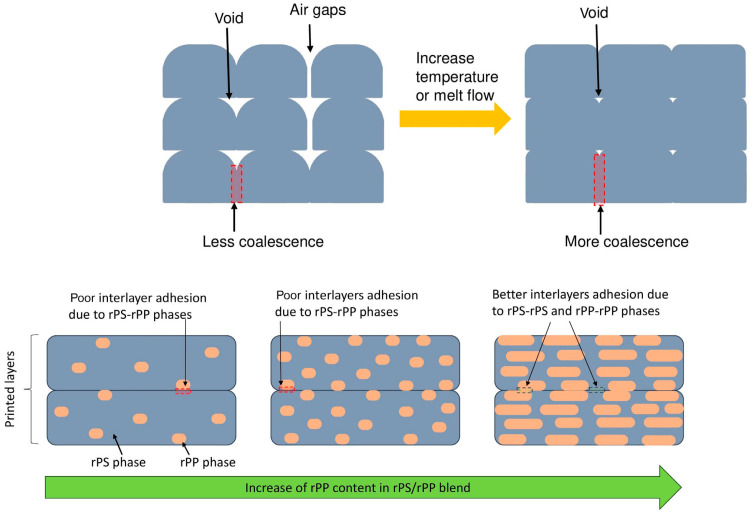
The Impact of Increasing Temperature and Content of Virgin Polypropylene on the Molecular Structure of Recycled Propylene [73].

In Figure 4, the impact of increased temperature and content of the virgin polypropylene on the molecular structure of recycled propylene was showcased. The insights showed that increasing the temperature and content led to better adhesion of interlayers.

However, recycled PET filaments also have exemplary mechanical properties when used as a standalone material for 3D printing. According to Oussai, Bártfai, and Kátai [21], continuous layering of PET wastes results in filaments with higher tensile strengths of up to 2.8% more than the strength of the virgin material. However, its hardness decreased by 6% and the shear strength increased by 14.7% compared to the virgin PET [21]. The tensile strength of recycled PET also reached a high of 43.15 MPa at a 3% elongation, suggesting that it is an ideal 3D printing material [21]. Likewise, Exconde et al. [80] assert that recycled PET has mechanical properties similar to virgin PET. Woern et al. [76] further established that recycled PET has no identifiable defects or adverse effects on the mechanical properties of the reprocessed filaments. The findings consistently show that recycled PET can make an alternative to virgin PET feedstock in 3D printing to promote circular economics and sustainability.

Similarly, filaments from recycled PLA demonstrated improvement in mechanical properties. Elumalai et al. [74] found that filaments from recycled PLA had improved tensile and impact strength to 25.66% and 32.16%, respectively. The water absorption rate of PLA also decreased by 89.96%. In contrast, Lanzotti et al. [81] established a decline in the strength of PLA following recycling. According to [81], the short beam strength of recycled PLA was 106 MPa with an allowance of 9 MPa compared to virgin PLA, which had a short beam strength of 119 MPa with an allowable strength of 6.6 MPa. Upon the second recycling, PLA had a short beam strength of 108 MPa, and 75 MPa following the third recycling [78]. The strength of PLA deteriorates with the frequency of recycling, indicating that it might not find a reliable long-term application. Anderson [45] aligned with [81] and showed that 3D printing filaments from recycled PLA have a 10.9% less tensile strength, a 6.8% higher shear strength, and a 2.4% lesser hardness than virgin PLA. The findings contradict evidence from Elumalai et al. [74] but align with findings by Lanzotti et al. [81] about the deterioration in the tensile strength of recycled PLA filaments. As a result, there is a likelihood of a decline in the mechanical strength of PLA following continuous recycling. The findings are further supported by evidence from Kumar et al. [64] that PLA loses 50 to 60% of its strength following the third recycling cycle. PLA recycled from virgin materials yields a 22% decline in mechanical strength compared to the original virgin PLA polymer. According to [64], only 85% of the strength is retained following first-time recycling. Similarly, an experiment conducted by Diego et al. [82] demonstrated that PLA loses viscosity by 15% for every recycling cycle. The change in viscosity after ten recycling cycles results in a 70% decline in ultimate strength, a 41% decrease in yield stress, a 38% decrease in Young’s modulus, and a 69% drop in fatigue resistance. Thus, repeated recycling of PLA might not be possible, given the deterioration in its mechanical properties. The implication is that the viscosity of the material is reduced with every recycling cycle, hence a decline in yield stress, ultimate strength, and fatigue resistance.

Nonetheless, an experimental study by Cisneros-López et al. [71] revealed that the strength of PLA can be enhanced by 88% by adding a chain extender to the recycled material. Likewise, Eren et al. [83] note that a higher polymer volume in the 3D printed material predicts higher compressive modulus and compressive strength, which are desirable mechanical properties for materials for tough applications. The findings align with evidence from Bergaliyeva et al. [63] that adding virgin PLA material during recycling of PLA yielded strengths of 52.61 MPa, representing an increase from 44.20 MPa for the virgin material. Adding virgin PLA material in recycled PLA is an integral process when producing 3D printing filaments with outstanding mechanical properties.

**Figure 5:**
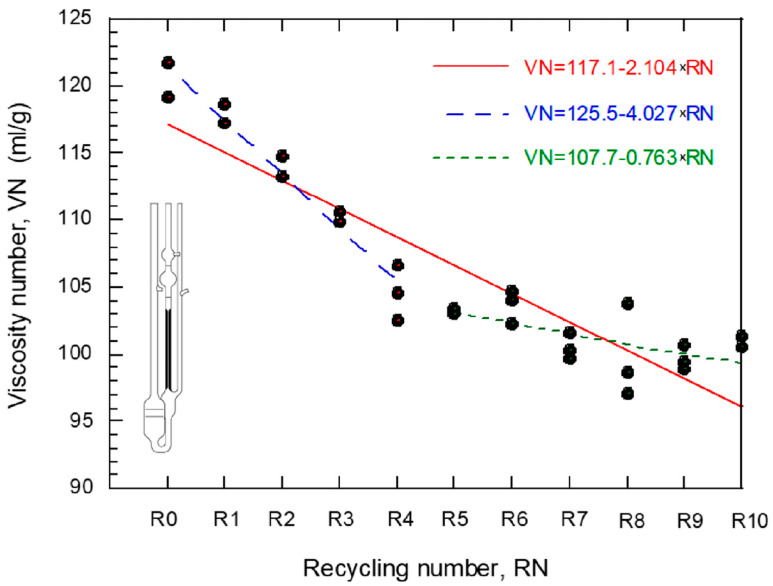
Change in Viscosity of Recycled Polymer at Different Recycling Cycles [82].

In Figure 5, the change in viscosity of recycled polymers at different recycling cycles is observed where an indirect relationship is reported. The increase in recycling cycles is observed to decrease the viscosity of the recycled polymers. 

Experimental tests performed with ABS recycled filaments also demonstrated variation in mechanical properties. ABS recycled at temperatures of up to 125 °C has a shorter radius of curvature. Accordingly, 3D-printed components from recycled ABS cannot be curved at a bigger radius because they are likely to fail. Similarly, the strength of recycled ABS was found to be lower than that of virgin ABS. According to Gaikwad et al. [77], recycled ABS filaments had 76% and 83% of their virgin materials’ breaking and tensile strength, respectively. Mohammed et al. [65] also acknowledged that recycled ABS’s strength is lower than virgin ABS’s because heat treatment lowers the molecular mass of ABS and reduces the molecular chains, making it weaker. Likewise, Bergaliyeva et al. [63] found that the molecular mass of PLA decreases upon extrusion recycling. Table 1 summarizes the variation in molecular mass for virgin and recycled PLA as observed in [63]. Therefore, recycled ABS is more likely to have poor mechanical properties than a combination of ABS, PLA, PET, ABS, and HDPE. However, according to Eren et al. [83], the orientation of the material during 3D printing determines its final mechanical properties. An experimental study by [83] revealed that horizontally printed polymers have higher compressive strength and compressive modulus. A high compressive modulus of about 1.8 GPa is achieved for 3D printed material with equidistant fiber reinforcement. For horizontal 3D printed polymers, the compressive modulus increases by 11.64%, while the compressive strength increases by 12.80% [83]. Therefore, the mechanical properties of recycled ABS, PET, and PLA can be improved through horizontal printing and reinforcement.

In Table 2, the variation in molecular mass for the virgin and recycled PLA is showcased, where an increase in weight ratio is observed with the recycled PLA.

### 3.6. Challenges of Recycling 3D Printed Plastic Wastes

Recycled 3D printing polymers are vulnerable to defects, including fiber misalignment and breakage. A weak fiber and matrix interface with uneven pressure during extrusion results in misalignment that has been found to lead to porosity [72]. Angles of curvature exceeding 120° and a turning radius of less than 5 mm account for the ease of breakage of 3D-printed polymer filaments. The inner periphery also develops lines of weakness with a decline in the radius of curvature and an increase in the angle of curvature [72]. Similarly, Atsani and Mastrisiswadi [68] established filaments from recycled polymers that easily bend, unlike virgin polymer filaments. Like [68], the authors of [71,72] found that internal defects are common in recycled polymers exposed to either extreme temperatures or UV light. Both [71] and Nagengast et al. [84] agree that the internal defects in recycled polymers render them functionally unreliable, and, therefore, they are disposed of as waste. These defects imply a limitation on using 3D printing as a plastic recycling method. The findings consistently affirm that 3D printing can also be a source of plastic waste, suggesting that 3D printing is not a sustainable method of managing plastic waste.

There are risks of a decline in the chemical and mechanical properties of the recycled polymers for every recycling cycle. A literature review demonstrates a likelihood of all polymers losing their strength with every recycling cycle and exposure to UV and high temperatures. Polymers undergo degradation by photolysis when exposed to ultraviolet light [75]. The synthesis of [75] and Nagengast et al. [84] indicates that the long-chain hydrocarbons break upon exposure to light or excess heat, rendering a polymer material brittle. Using an experimental research design involving exposure of polymers to UV light, [75] found that 3D printed filaments deteriorate on exposure to ultraviolet light, rendering them mechanically weaker. Also, the strength of PET deteriorates with the increase in the frequency of recycling [81]. According to Ji and Jung [67], polypropylene polymers cannot be recycled multiple times because their mechanical properties deteriorate with every subsequent hot extrusion at temperatures of 200 °C. Likewise, Nagengast et al. [84] established that 3D-printed PLA loses its mechanical strength following thermomechanical treatment. Degradation was also noticed in its rheological and dimensional properties after repeated recycling for up to three cycles.

The outcomes corroborate evidence from Bergaliyeva et al. [63] that the mechanical strength of PLA significantly reduces for every recycling cycle. Cisneros-López et al. [71] further add that the recyclability of PLA to make 3D printing filaments might be limited because filaments produced from recycled PLA cannot be repeatedly used in making functional components due to a rapid decline in their mechanical strength following hot melt extrusion. The same applies to polypropylene and polystyrene, which were found to lose their tensile strength following repeated extrusion [66]. Recycling could lead to a weak internal structure and render them susceptible to breakage upon loading [77]. Pricop et al. [78] and Zhu et al. [39] agree that repeated recycling of 3D-printed plastics is impossible due to the shortening of hydrocarbon chains, leading to a weaker internal structure. Figure 5, as shown below, demonstrates the behavior of polymers after different recycling cycles. The figure demonstrates a general decline in strength with an increase in the number of recycling cycles. According to Zhao et al. [85], repeated 3D printing of PLA leads to the deterioration of the mechanical properties of most polymers, including PLA and PET. Thus, repeated recycling of 3D printed products might not be sustainable in the long run, suggesting a likelihood of accumulation of 3D printed polymer wastes in the surroundings. Likewise, Rigon et al. [86] noted that the mechanical properties of Polypropylene (PP) decline after recycling and extruding them into filaments. The elastic modulus decreases by 20%, while the elastic modulus decreases by 12.9% with every subsequent recycling cycle [86]. Accordingly, the materials cannot be relied upon after more recycling cycles. The findings also suggest a likelihood of 3D printing resulting in higher amounts of plastic waste from components that have lost their functional integrity following deterioration.

**Figure 6:**
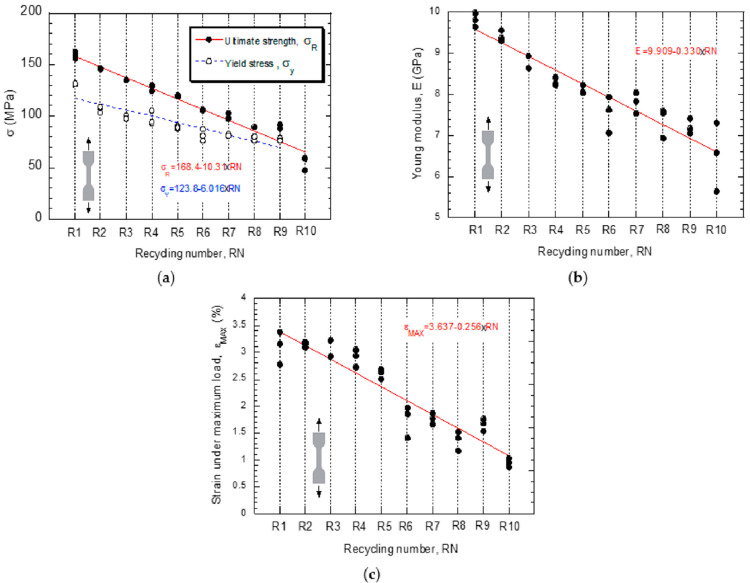
Experimental Evidence Demonstrating a Decline in Strength of Recycled Polymers [82] for different tested parameters (**a**–**c**).

In Figure 6, insights show that there is a decline in the strength of recycled polymers with an increase in the recycling cycles. The strength is measured using yield stress, ultimate strength, and the strain under maximum load.

Furthermore, given that some 3D-printed plastics are not recyclable, there has been an alarming rise in 3D-printed plastic waste due to the rapid growth in 3D printing technology [79]. Thermosets used in 3D printing are considered dangerous to the environment since they are non-biodegradable and contain a high number of carbons that, when oxidized, lead to the formation of carbon dioxide. This harmful greenhouse gas has accelerated climate change [79]. Likewise, Xu et al. [38] established that 3D printing will likely lead to a rise in 3D-printed plastic waste because most materials are non-biodegradable. In addition, the synthesis of [38,79] reveals an agreement that the properties of recycled 3D printing plastics deteriorate over time, suggesting that they often become functionally obsolete and are dumped in the surroundings. In parallel, Beltrán et al. [44] argue that the heating process during the recycling of plastic wastes reduces polymer chains and increases the degradation rate of recycled polymers. Thus, there are higher risks of the materials accumulating and posing more environmental danger. As such, an alternative biodegradable 3D printable resin must be sought.

### 3.7. Local and Distributed Recycling and their Environmental and Economic Sustainability

A review of select studies among the thirty-nine articles demonstrated that distributed recycling has the potential to reduce energy consumption, carbon dioxide emission, and the expense of recycling plastics. Kreiger et al. [87] established that distributed recycling of HPDE uses low embodiment energy and reduces carbon emissions. For instance, an experiment conducted by [87] revealed that distributed recycling requires only 8.74 MJ/Kg of energy, while centralized recycling consumes 79.67 MJ/Kg of energy in processing virgin HDPE filament. Likewise, Santander et al. [27] determined that distributed recycling involves a closed-loop approach comprising small coordinated units characterized by low energy consumption. Despite the complexity associated with the method, findings from [27] demonstrate that it is characterized by low energy consumption and low carbon dioxide emission, which render it economical and environmentally friendly. Unlike the conventional model summarized in Figure 6, the distributed recycling model does not involve waste collection from different sources and transportation to a central recycling point, making it less energy-intensive and economically sustainable [27]. Likewise, Zhong and Pearce [69] found that distributed recycling reduces embodied energy by half and minimizes the cost of consumer products made from plastics recycled by the distributed recycling technique. Consequently, it is an economical and environmentally friendly technique for recycling plastics into 3D printing feedstock, for instance, for agricultural applications.

**Figure 7:**
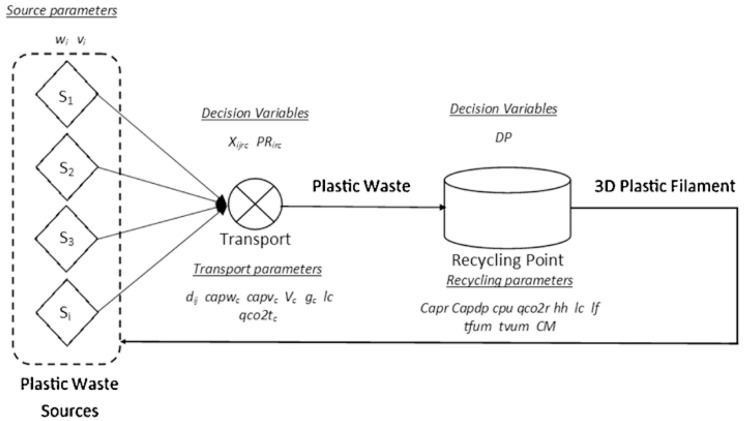
Conventional Recycling Practices Entailing Waste Collection and Transport to Recycling Point [28].

In Figure 7, the recycling practices are summarized as processes that involve transportation of the waste to the recycling point to generate 3D plastic filaments. 

Local recycling was also found to be viable with distributed recycling. Santander et al. [27] and Kreiger et al. [87] agree that distributed recycling can be localized to reduce logistical costs and foster plastic waste recycling at production points. Like [27,87], Zhong and Pearce [69] further acknowledge that distributed recycling allows for local recycling of wastes with the benefits of recycling wastes and using them as feedstock at the point of production. Therefore, distributed and local manufacturing reduces energy and transport costs, rendering the techniques economical and viable on a large scale. The process of conventional recycling entailing plastic waste collection and transport is summarized in Figure 6. Following an experiment to determine the strength of PLA recycled using the distributed recycling technique, Beltrán et al. [44] established that feedstock from distributed recycling has mechanical properties that compare to those of virgin PLA. Therefore, the evidence consistently affirms that distributed recycling in local settings is a feasible, economically viable, and environmentally friendly strategy for recycling waste.

### 3.8. Application of 3D Printing in Agriculture

**Figure 8:**
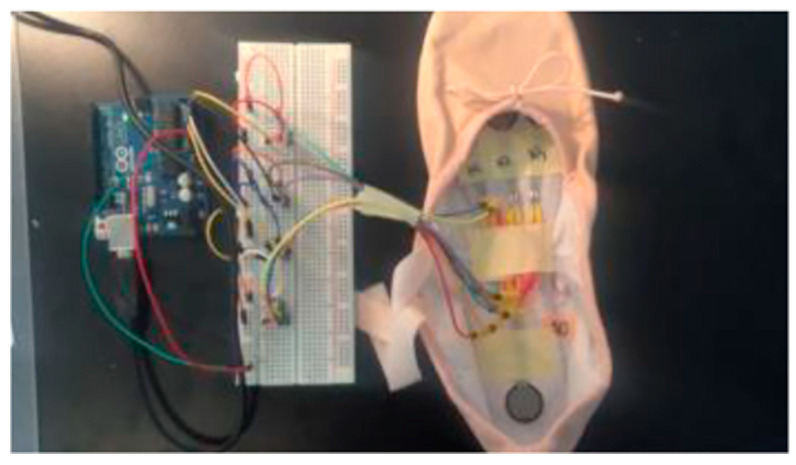
Manufacturing process for recycled polymers [65].

The focus on agricultural plastics follows the local production of farm wastes and the likelihood of recycling them through distributed recycling techniques from where they are produced, particularly farms. Given the adaptability of agricultural plastic waste management in distributed and local recycling, functional agricultural plastic products can also be locally produced through 3D printing using filaments from recycled plastics. Maraveas et al. [88] and Maraveas [89] agree that increased agricultural plastic waste results from their limited durability. Similarly, a Lifecycle Assessment (LCA) of plastics by Vidakis et al. [90] and Mohammed et al. [65] revealed that less durable plastics contribute to the rapid generation of plastic waste. Regardless, recycling them has increased the potential to make reliable, usable components. Figure 8 showcases the manufacturing process for recycled polymers adopted in [65].

In Figure 8, the manufacturing process for the recycled polymers is showcased where granulation, filament extrusion and spooling, and FDM printing are observed.

According to Maraveas [91], anti-hail, windbreak, and anti-insect plastics are produced from 3D-printed filaments from plastic wastes. 3D-printed wastes for HDPE, PVC, and PE are the material of choice for agricultural applications due to their high tensile strength compared to weak bioplastics that often interfere with the lifecycle of natural predators like spiders [92]. The evidence demonstrates the viability of recycling agricultural plastics, primarily through distributed local recycling, which has the advantages of low carbon emissions and lower recycling costs, given that the wastes do not need to be transported. Likewise, Santander et al. [27] and Kreiger et al. [87] note that functional products can be produced locally in distributed manufacturing. As a result, plastics produced from farms can be recycled by distributed recycling within the farms, and the filaments can be 3D printed into helpful plastic products for agricultural applications.

However, there might be challenges in the frequent recycling of agricultural plastics. For instance, Sun et al. [75] and Nagengast et al. [84] found that recycled plastics rapidly deteriorate on exposure to UV light, indicating that they are less durable. Regardless, all wastes can be recycled within farms with locally distributed recycling. There is also potential to reduce waste by improving the durability of the plastics made from recycled feedstock. Experiments by Yousif et al. [93] and El-Hiti et al. [94] suggest that during 3D printing, polymers can be stabilized against UV light to reduce their vulnerability to photolysis due to exposure to UV light. The findings consistently demonstrate that 3D printing can make more potent and durable plastics for agricultural applications. The durability aspect is also accompanied by improved agricultural application functions, such as antimicrobial activity and effective light transmittance. Maraveas [92] found that recycled filaments produce shed nets with outstanding heat resistance and anti-oxidation, rendering them more effective and durable in agricultural applications. Similarly, Maraveas [95] found that 3D printing as an additive manufacturing technique improves the UV transmittance of greenhouse covers with slow insecticide release and improved anti-microbial properties. Therefore, adding UV blockers to filaments from plastic wastes can enhance the properties of plastics produced through locally distributed recycling with the potential of reducing recycling costs while promoting environmental sustainability by reducing carbon emissions and consuming high amounts of energy.

### 3.9. Future Directions and Further Research in the Use of 3D Printing for Recycling Plastics

Future development and research should primarily regard improving the strength of recycled polymers and reducing their vulnerability to photolysis. From Figure 5, Diego et al. [82] demonstrated that the strength of PLA declines significantly with every recycling cycle. Similarly, the elastic modulus of polypropylene decreases by 20% and the elastic modulus decreases by 12.9% with every recycling cycle [87]. The results corroborate those from Sun et al. [75] and Nagengast et al. [84] that the strength of polymers declines following exposure to UV light, leading to the shortening of the long-chain hydrocarbon bonds in the polymeric structure. Likewise, Gaikwad et al. [77] acknowledged that recycled ABS has less than 90% of the strength of virgin ABS. Some of the critical procedures that have proved reliable in minimizing the deterioration of polymers include combining or fusing the polymers and adding anti-photolysis agents to prevent the degradation of plastics exposed to UV light. Zander et al. [36] found that a combination of PET, PLA, and ABS results in 3-D printing filaments with exemplary mechanical properties, including a higher tensile strength of up to 35 Mpa. On the other hand, Mohammed et al. [65] stipulated that a combination of 90% ABS and 10% HDPE produces filaments with consistent 3D prints and mechanical strengths up to 20% higher than the respective parent virgin polymers. Therefore, a combination of polymers can help enhance their strength and reduce their deterioration rate. However, factors other than the strength of polymers should also be studied to provide a comprehensive understanding of the different plastic waste types that can yield reliable 3D printing feedstock. On the other hand, a future development to addressing photolysis cited by Sun et al. [75] and Nagengast et al. [84] as a setback to the durability of recycled polymers concerns the addition of anti-photolysis agents, which inhibit degradation when exposed to light. According to El-Hiti et al. [94], a PVC film blended with Schiff, Thiadiazole Moiety, and Nickel Chloride is resistant to photolysis. Yousif et al. [93] also found that the weight and surface morphology, mainly the smoothness of polystyrene, decline following irradiation with UV light. However, after adding Schiff bases, polystyrene stabilized against UV radiation [93]. Nonetheless, factors other than photolysis should be studied to establish how they affect the reliability of recycled polymers. The approach should primarily entail determining how fusing and doping with agents, such as Schiff, affect recycled polymers’ chemical and mechanical properties.

## 4. Discussion

The study aimed to explore and review the literature and synthesize evidence on how 3D printing is used in recycling and its application in the production of agricultural plastics. In fulfilling the research aim, the study focused on establishing methods used in recycling plastics for 3D printing, the properties of 3D printing feedstock from recycled plastics, the advantages of 3D recycled polymers, applications of 3D printing in agriculture, the limitations of the recycled polymers for 3D printing, and the future directions in using 3D printing for recycling plastics. The discussion section focuses on a comprehensive discussion of findings to establish consistency in evidence regarding using 3D printing as a strategic method for recycling polymers.

### 4.1. 3D Printing as a Plastic Recycling Method and Its Advantages and Limitations

The study findings demonstrated that 3D printing is a recycling method for recycling plastics. The results showed that the availability of recycling methods, such as FPF, HME, and FDM, has promoted the recycling of plastics to make 3D printing filaments. Cisneros-López et al. [71], Beltrán et al. [44], and Zhang, Chen, and Yang [72] demonstrated that HME is a reliable method of turning plastic wastes into 3D printing filaments. Elumalai et al. [74] established that FDM is a reliable method of turning plastic wastes into molten material molded into 3D printing filaments. Similarly, findings from Stefaniak et al. [96] and Shiferaw and Gebremedhen [97] show that recycling plastics into 3D printing filaments is made possible by the availability of reliable recycling methods, such as HME and FDM. According to Mikula et al. [17], these techniques have allowed for rapidly converting plastic waste into helpful feedstock for 3D printing. The findings consistently demonstrate that the availability of methods of turning plastics into 3D printing filaments has allowed the continued utilization of 3D printing as a reliable plastic recycling method. Differently, findings from Lee et al. [98] and Morales et al. [99] highlight that the advent of rapid prototyping and reverse engineering has promoted the recycling of plastics into 3D printing filaments. Evidence from Maines et al. [79] and Pricop et al. [78] is consistent with evidence from Mikula et al. [16] that rapid prototyping has allowed for the creation of 3D printed models designed with CAD technologies. According to findings by Polline et al. [100], rapid prototyping allows for the creation of objects of different models and sizes with the utilization of cheap 3D printing feedstock from recycled plastics. The findings consistently show that 3D printing is a reliable method of recycling plastics, given the availability of supporting technologies, such as HME, FDM, and rapid prototyping. All available plastic wastes can be assessed, and their appropriate applications can be defined for their utilization in 3D printing procedures. 3D printing filaments from recycled plastic wastes will likely reduce plastic waste in the surroundings and address their overall adverse environmental effects.

Further evidence demonstrated that the need for cheap 3D printing filaments has promoted the collection of plastic waste from landfills and oceans for recycling. Vones et al. [70] and Silva et al. [101] established that marine firms recycle plastic waste from oceans to make 3D printing filaments. Findings from Gil Muñoz et al. [102] also showed that 3D printing has become influential in the collection and recycling of plastic wastes from safety gear firms to make 3D printing feedstock. The evidence is consistent with results from Mishra, Negi, and Kar [47] and Mikula et al. [17] that the advent of technologies that support recycling plastics into 3D printing filaments has promoted plastic recycling and the elimination of plastic wastes from the surroundings. The approach has since reduced the accumulation of plastic waste in landfills and marine environments. According to the findings, using recycled plastic for 3D printing filaments is a sustainable idea that can help avert the adverse effects of plastics in the environment. The sustainability of 3D printing is also demonstrated by reducing overreliance on virgin polymer filaments as feedstock. According to results from Santander et al. [27] and Sun et al. [75], recycled 3D printing filaments are economical and sustainable given that their processing does not involve consuming large amounts of energy or the emission of high volumes of greenhouse gases. The results corroborate evidence from Kreiger et al. [87] that HDPE-distributed recycling uses low embodiment energy and reduces carbon emissions. Madhu et al. [11] and Cunico et al. [50] note that recycled polymers are cheaper compared to virgin materials used in producing 3D printing filaments, given that they do not have intensive energy requirements nor emit harmful greenhouse gases that contribute to atmospheric pollution and increase risks of global warming. Thus, making 3D filaments from recycled polymer is economical and reduces carbon dioxide emissions.

The fact that 3D printing has promoted the use of recycled polymer filaments suggests that it is likely to promote the circular economy, which is crucial in achieving the net-zero objectives of reducing carbon emissions. According to research outcomes from Maines et al. [79], Gaikwad et al. [77], Zander et al. [36], and Zhong and Pearce [69], 3D printing has promoted the use of recycled plastics as the primary raw materials. Evidence from Gil Muñoz et al. [102] that the use of plastic consumer wastes to make 3D printing filaments suggests the circular nature of the present economy, in which waste products are used as crucial inputs or raw materials in subsequent processes. The findings are consistent with evidence from Garmulewicz et al. [103] and Pavlo et al. [104] that 3D printing is an enabler of the circular economy because it allows heat treatment of plastic wastes to convert them into helpful feedstock for 3D printing. According to findings from a literature review conducted by [104] and Nadagouda, Ginn, and Rastogi [105], 3D printing has resulted in a closed-loop supply chain in which waste materials are being used as feedstock for 3D printing processes to produce functional components. Further consistency in evidence is demonstrated by results from Sanchez et al. [106] and Mikula et al. [17] that 3D printing has enabled distributed recycling through additive manufacturing, which allows for converting plastic wastes to filaments that serve as feedstock for 3D printing processes. The plastic recycling approach renders 3D printing a reliable method for recycling plastic polymers and turning them into meaningful parts that can still be recycled. Arguably, the available plastic waste can be adequately managed if 3D printing is embraced on a broader scale.

However, a significant limitation to the use of 3D printing regards the rapid degradation of 3D printed products made using recycled polymers. The mechanical strength of the recycled 3D printing filaments declines with every subsequent recycling cycle. According to findings by Sun et al. [75] and Nagengast et al. [84], exposure of polymers to UV light increases their degradation because the light breaks long-chain hydrocarbon bonds, rendering them brittle. Evidence from Pinho, Amaro, and Piedade [2] and Hidalgo-Carvajal et al. [42] also shows that the mechanical strength of polymers declines following repeated heat treatment and exposure to UV light, which breaks down their long-chain bonds and reduces their molecular mass, rendering them weaker. Arguably, the integrity of recycled polymers declines significantly over time, indicating that they might not be reliable for extended periods. For instance, findings of an experimental study involving six plastic recycling cycles by Di and Yang [43] demonstrate that Young’s modulus, the strain and stress at the breakpoint, rheological properties, and hardness decrease for every recycling cycle. As such, the materials cannot be relied upon in the long run, given that their functional integrity can rapidly decline, rendering them risky. Accordingly, in the long run, an alternative approach to strengthening the materials should be determined to help enhance the durability of products from recycled 3D printing filaments.

The systematic review also revealed a consistent decline in the mechanical strength of polymers such as PET, ABS, PLA, and PVC with every recycling cycle, except for the shear and tensile strength. Findings from experiments on PET and PLA by Oussai, Bártfai, and Kátai [21] and Anderson [45] demonstrate an increase in shear and tensile strength of recycled polymer filaments over the respective virgin polymer filaments. The findings contradict evidence from Beltrán et al. [44] that repeated recycling of polymers does not alter their mechanical properties. Concerning ABS results from Cress et al. [46] and Mishra et al. [107] suggest that the material properties do not change regardless of the repeated heat treatment during recycling and making 3D filaments. Rigon, Ricotta, and Meneghetti [48] and Atakok, Kam, and Koc [41] also found that 3D printing filaments from recycled ABS have properties not found in virgin filaments. The contradictions in evidence suggest further research is necessary to capture the actual variations in mechanical and chemical properties of recycled polymers in 3D printing filaments. However, evidence of the consistent decline of mechanical properties of polymers following recycling is demonstrated by experiments carried out by Sun et al. [75], Nagengast et al. [84], and Bergaliyeva et al. [63]. On the other hand, Beltrán et al. [44], Cress et al. [46], and Mishra et al. [107] relied on secondary evidence to ascertain the changes in the mechanical properties of recycled polymers. Therefore, variations in the findings can result from differences in research designs and data analysis approaches adopted. Accordingly, future studies should be based on a standard and common methodology to ensure consistent findings and provide reliable study outcomes about the mechanical behavior of 3D printing filaments from recycled polymers.

Nonetheless, the fact that recycled 3D printing materials deteriorate faster and are limited to specific recycling cycles suggests they pose a further risk to environmental sustainability. Results from Beltrán et al. [44] show that repeated recycling of polymers hastens their degradation. Evidence from Sun et al. [75] and Nagengast et al. [84] also highlights that photolysis reduces the integrity of recycled polymers due to the rapid breakdown of molecular bonds and the shortening of the hydrocarbon chains. Per Xu et al. [35] and Maines et al. [79], deteriorated 3D printing products cannot be further recycled and thus pose a menace to the environment because they are not biodegradable. The findings by Xu et al. [38,79] are consistent with findings from Hong et al. [108] and Patti et al. [109] that the end-of-life of 3D printed products from recycled polymers is not often defined because the deteriorated material cannot be further recycled. Thus, there are higher risks of the materials accumulating in the surroundings, posing more danger to the environment. According to findings from Atakok, Kam, and Koc [41], there is a likelihood of an increase in 3D printed wastes in the surroundings because of a lack of reliable techniques for further recycling 3D printing polymers. Accordingly, there are risks of accumulation in 3D printing wastes with negative implications on the land and marine life. Consequently, alternative approaches must be sought to foster continued recycling of general plastic wastes and those from 3D printing applications. For instance, evidence from El-Hiti et al. [94] and Yousif et al. [93] shows that filaments made from recycled polymers can be stabilized to resist degradation following exposure to UV light. Likewise, findings from Allen and Edge [110] and Ambrogi et al. [111] demonstrate a likelihood of stabilizing polymers to resist deterioration following exposure to heat and UV light. Accordingly, new approaches to enhancing the properties of recycled polymers for 3D printing applications must be sought to address the likely challenges stemming from producing 3D-printed wastes that cannot be managed through recycling.

### 4.2. 3D Printing in Agriculture

3D printing has found application in managing agricultural plastic wastes to make useful plastic products for farming applications. Evidence from Maraveas et al. [88] and Maraveas, Bayer, and Bartzanas [112] consistently shows that 3D-printed Nano-materials exhibit exemplary toughness because they are reinforced and multi-layered for high strength. The examination of the studies [88,112] indicates that the feedstocks used in the 3D printing comprised of clean thermoplastics that were used in the manufacture of greenhouse covers and other agricultural applications such as shade nets and food packaging. Additionally, the feedstocks used included PLA and ABS materials that were non-biodegradable polymer filaments [112]. The 3D printing technology is also linked to the development of PLA materials with controllable shape memory and self-deformation, which are vital to the growth and development of smart agriculture [113]. The findings consistently show that 3D printing is more likely to revolutionize agriculture by increasing the recycling of agricultural plastic wastes, producing more durable agricultural plastics, and enhancing agricultural production by developing smart agricultural plastics.

The study findings demonstrated that distributed recycling has the potential to reduce embodied energy, carbon dioxide emissions, and overall costs of recycling plastics. Findings by Kreiger et al. [87] and Santander et al. [27] are consistent with evidence from Hunt et al. [1] and Lee et al. [95] that distributed recycling reduces energy consumption and carbon dioxide emissions during recycling. Likewise, Sanchez et al. [106] and Mikula et al. [17] argue that distributed recycling has allowed for the production of 3D printing feedstock from plastic wastes at their production point, reducing transport costs and energy consumed in conventional global recycling techniques. The fact that Beltrán et al. [44] and Pavlo et al. [104] successfully used the distributed recycling technique with local manufacturing to produce products with properties comparable to virgin polymers demonstrates the feasibility of the use of the method in recycling agricultural products and making functional components within the farm. Accordingly, the technique remains economically and environmentally viable in recycling agricultural plastic wastes and making functional components within farms.

### 4.3. Future Directions in the Use of 3D Printing for Recycling Plastics Concerning Lifecycle

The findings demonstrated that future trends in recycling plastics center on addressing their rapid deterioration due to photolysis and loss of mechanical strength with every subsequent recycling cycle. According to the systematic review outcomes, recycled polymers can be stabilized by combining various polymers with additives such as nickel oxide for stability against UV light. Findings from Zander et al. [33] show that a combination of PET, PLA, and ABS results in 3-D printing filaments with a tensile strength of up to 35 times greater than that of individual recycled polymers. On the other hand, Mohammed et al. [65] show that 90% ABS and 10% HDPE yield filaments with 20% higher tensile strength than the respective parent virgin polymers. Findings by [65] and Zander et al. [36] are consistent with results from Zhang et al. [114] that doping polymers with either virgin material or other types of polymers can substantially enhance their mechanical properties. According to research outcomes from Jia et al. [115], the fusion of polymers during recycling or heat treatment leads to the formation of polymers with superior chemical and mechanical properties. Arguably, combining the materials helps offset the weaknesses of the individual materials, leading to enhanced material properties. As a result, combining different polymers during recycling can help address the loss in strength of polymers following repeated recycling. Likewise, findings by El-Hiti et al. [94] and Yousif et al. [90] are consistent with evidence from Ahmed et al. [116] and Li [117] that blending polymers with additives such as Schiff, Thiadiazole moiety, and Nickel Chloride reduces risks of photolysis and rapid deterioration. Findings by Amza et al. [118] also demonstrate that UV light stabilizers or blockers, such as Benzotriazoles and benzophenones, reduce the vulnerability of polymers to photolysis. Accordingly, photo stabilization should be pursued as a reliable technique for preventing the rapid degradation of recycled 3D printed polymers and reducing dumping and accumulation in the surroundings.

### 4.4. Limitations

The study significantly demonstrated the usability, advantages, and limitations of 3D printing in recycling plastics. However, there are significant limitations concerning the use of secondary evidence in synthesizing findings in the present study. For instance, the contradictory evidence about the changes in the mechanical properties of recycled polymers for every subsequent recycling cycle demonstrates the need for primary evidence following experimentation with each recycled polymer to provide first-hand evidence about the mechanical properties of polymers following recycling. Furthermore, errors in the findings of the 39 reviewed studies might have been incorporated into the present review. Nonetheless, the rigorous quality assessment method ensured that only credible sources were included in the review. Accordingly, the study evidence remains authentic and reliable within the present research context.

### 4.5. Recommendations

The study demonstrated that the degradation of recycled polymers and the loss of their functional integrity are significant limitations to the reliability of 3D printing as a plastic recycling method. Thus, further research should be conducted to synthesize extensive evidence on enhancing the toughness of recycled polymers against extreme temperatures and UV light. For instance, methods of stabilizing recycled polymers for 3D printing should be sought to help enhance the durability of the recycled polymers and reduce the likelihood of accumulation of 3D printed wastes in the surroundings. Furthermore, new techniques and approaches to additive manufacturing must be sought to help enhance recycled polymers’ mechanical and chemical properties. For example, the findings demonstrated that a combination of polymers, such as PLA, PVC, HDPE, PET, and ABS, enhances the strength of the resultant polymer material. Therefore, a further assessment of the various methods of combining the polymers should be sought to promote the production of recycled polymer filaments with outstanding mechanical properties.

Future studies on using 3D printing for recycling plastics should employ a primary longitudinal research methodology to provide first-hand evidence, for example, on the changes in the chemical and mechanical properties of recycled polymer filaments. Experiments should be conducted over an extended period or at different recycling intervals to confirm how the properties of recycled 3D printing filaments change over time. The materials can be 3D printed, used, and recycled again to verify their behavior under different loading conditions at respective recycling cycles. Experiments can also be conducted to compare the properties of a combination of recycled polymer wastes or photo-stabilized recycled polymers and the individual recycled wastes that have no additives to confirm the variation in mechanical and chemical properties of the materials at different recycling frequencies and loading or exposure.

## 5. Conclusions

The study aimed to explore the use of 3D printing in recycling plastics. The research aim was fulfilled by meeting four main research objectives: finding out the methods and parameters in recycling plastics through 3D printing, investigating the benefits and limitations of using 3D printing for recycling/re-production, identifying future directions regarding the future of the use of 3D printing for recycling/re-production, and investigating the beneficial applications, especially on low recycling categories like agricultural plastics. The first research objective was met by demonstrating that plastic wastes are recycled into 3D printing filaments through diverse methods in a local recycling setup. The use of methods such as HME, FPF, and FDM which preprocess the plastic waste when creating printing raw material was identified in local recycling setups where only small quantities were considered. However, in a distributed or large-scale system, the FPF and FDM processes were not considered viable.

Some materials recycled into respective 3D printing filaments include PLA, PET, PVC, LLDPE, HDPE, PP, and ABS. The resultant materials have properties comparable to their parent material with margins of up to 80%. However, the material properties, including tensile strength and modulus of elasticity, decline for every recycling cycle. Nonetheless, in line with the second research objective, the systematic review revealed that recycling plastics to make 3D printing filaments has reduced over-reliance on virgin polymers for 3D printing. Processing virgin polymers during 3D printing also leads to higher energy consumption and the production of carbon dioxide that is harmful to the ozone layer. The availability of 3D printing methods such as rapid prototyping and effective modeling technologies like CAD has increased the use of recycled polymer filaments in 3D printing. The approach has resulted in collecting and recycling plastic waste from landfills and oceans. Accordingly, 3D printing has become a reliable approach to managing plastic waste. However, the rapid degradation of the recycled polymers and their loss of mechanical strength following repeated recycling demonstrates a likelihood of the method being less sustainable in the long run. The technique will likely lead to a rise in 3D printed plastic waste because most obsolete 3D printing materials are non-biodegradable. Recycling and degrading 3D-printed plastics produce carbon dioxide, the most common greenhouse gas with dangerous effects on the ozone layer. Thus, there are higher risks of the materials accumulating in the surroundings, posing more danger to the environment. Accordingly, methods, such as adding UV blockers and virgin material to recycled wastes, should be adopted to enhance the durability of the recycled 3D printed products. Regardless, 3D printing has found prominent use in recycling agricultural plastics and making durable and smart agricultural plastics capable of adjusting properties, form, and shape in response to weather conditions. Evidence shows that distributed and local recycling techniques reduce carbon emissions and energy consumption in plastic processing, leading to feedstock production with mechanical properties comparable to virgin polymers. It can be adopted in recycled agricultural plastics and manufacturing functional products within farms. However, regarding the third research objective, future directions concerning 3D printing should involve stabilizing 3D printing recycled polymers to make them tough to resist degradation and deterioration in their desirable properties, for instance, mechanical strength and chemical resistance. Methods such as UV blocker doping and fusion of different types of polymers to make 3D printing filament should be embraced to make more durable 3D printing filaments from recycled polymers. Production of more rigid and durable 3D printing filaments can further enhance plastic waste management by minimizing the disposal of 3D printed waste. Furthermore, there is limited evidence about the recycling of agricultural plastics. The aspects should mainly feature in further research towards providing robust mechanisms by which agricultural plastics can be recycled into 3D printing filaments and subsequent remodeling to make other usable agricultural products, for example, shed nets. Overall, the study demonstrated that modern technologies such as 3D printing could be leveraged to promote the circular economy where similar plastics are reproduced following the recycling process of virgin plastics. However, where recycling generates other non-comparable plastics, this promotes resource efficiency and diverts disposal to landfills. As a result, recycling leads to improved environmental sustainability and lower carbon emissions hence promoting the attainment of net-zero targets.

## Figures and Tables

**Table 1:** Methods used in the reviewed studies.

Method	Number of Studies
Case study	1
Experimental quantitative	33
Experimental mixed methods	6

**Table 2:** Variation in Molecular Mass for Virgin and Recycled PLA [63].

Sample Code	Weight Ratio (%)	
	Virgin PLA	Recycled PLA
V100R0	100	0
V75R25	75	25
V50R50	50	50
V25R75	25	75

## Data Availability

Not available.

## Appendix A. Literature Matrix

**Table A1:** Literature Matrix.

Source	Nature of the Study/Methodology	Materials	Results	Relevance
[72]	A quantitative study with an experimental design.	PLA, ABS, PET, and PP	3D printed polymers are vulnerable to defects, including fiber misalignment and breakage. A weak fiber and matrix interface with uneven pressure during extrusion results in misalignment that has been found to lead to porosity. Angles of curvature exceeding 120 degrees and a turn radius of less than 5 mm account for ease of breakage of 3D printed polymer filaments. The inner periphery also develops lines of weakness with a decline in the radius of curvature and an increase in angle of curvature.	The study demonstrates the defects likely to occur in recycled 3D printing polymers.
[73]	A quantitative study with an experimental design.	PLA	The radius of curvature decreases as the material is heated at gradually increasing temperatures. ABS and PLA exhibit the properties at temperatures of about 125 degrees centigrade. Therefore, the 3D printed recycled polymers cannot be curved at bigger radius because they are likely to fail or break.	The findings of the study contribute to an understanding of the nature of defects and limitations likely to occur in the use of 3D printing materials. Also, Mendenhall and Eslami (2023) provided in-depth insights into the expected mechanical properties of ABS and PLA filaments made from respective polymer wastes.
[83]	A quantitative study with an experimental design.	ABS, PLA, PET, and PP	Horizontally printed polymers have higher compressive strength and compressive modulus. A high compressive modulus of about 1.8 GPa is achieved for 3D printed material with equidistant fiber reinforcement. For horizontal 3D printed polymers, the compressive modulus increases by 11.64%, while the compressive strength increases by 12.80%. A higher polymer volume in the 3D printed material predicts higher compressive modulus and compressive strength, which are desirable mechanical properties for materials for tough applications.	The study provides a strategic approach to strengthening recycled 3D printing materials. The study therefor fosters an understanding of the different approaches by which polymer wastes can be used despite being linked to particular defects.
[77]	A quantitative study with an experimental design.	ABS	Electronic waste plastics mainly polycarbonates were transformed into 3D printing filaments. The materials had 76% and 83% breaking strength and tensile strength of their virgin materials such as ABS. 3D printed filaments from e-waste also exhibited high flexibility compared to virgin filaments. Using the 3D filaments from e-waste as feedstocks instead of virgin polymers accounted for a 28% decline in carbon emissions. However, periodic recycling could lead to a weak internal structure and render them susceptible to breakage upon loading.	The study confirmed that 3D printing can be used in addressing the electronic plastic waste menace, demonstrating its reliability in recycling polymers and reducing the carbon footprint.
[36]	A mixed-methods study with an experimental research design and mechanical property analysis of recycled 3D printing polymers.	ABS, PET, and PLA	Consumer-grade plastics and polymers are considered a sustainable feedstock for 3D printing. Given that separation of e-waste is often challenging, blended 3D polymer filaments are produced by PET, PLA, and ABS together. Resulting filaments exhibit exemplary mechanical properties, including higher tensile strength of up to 35 Mpa. Glass transition for the materials also rose, suggesting that the materials could not easily melt at elevated temperatures. The aspect renders 3D printed filaments from blended polymers thermal resistant.	The study demonstrated the feasibility of recycling consumer plastics by 3D printing. The production of usable components through 3D printing using waste plastics as feedstock demonstrates the sustainability of the process.
[27]	A mixed-methods experimental study with thematic and descriptive analysis.	Plastic wastes	3D printing of plastic waste has helped in creating a circular economy. The 3D printing technique for plastic wastes has proven economically and environmentally viable because it reduces over-reliance on virgin material that are expensive in comparison to plastic wastes and minimizes accumulation and incineration of plastics in the surrounding. Overall, it reduces the over-dependence on virgin plastics for production of functional products, an aspect that is acknowledged for reducing carbon emissions and emptying landfills that pose danger to soil micro-organisms.	The study affirmed the sustainability of 3D printing. From the study, plastic wastes can be turned into 3D printing filaments, thus preventing their accumulation in the surrounding. Therefore the study confirms that 3D printing is a reliable method of recycling polymers.
[91]	A quantitative study with an experimental design.		Anti-hail, windbreak, and anti-insect plastics are produced from 3D printed filaments from plastic wastes. 3D printed wastes for HDPE, PVC, and PE are the material of choice for agricultural applications due to their high tensile strength in comparison to bioplastics that are weak and often interfere with the lifecycle of natural predators like spiders.	The study demonstrates how 3D printing can be used to make agricultural plastics with outstanding properties for durability and reduced plastic wastes as a result of rapid deterioration.
[76]	A quantitative study with an experimental design.	PET and PP	Fused Particle Fabrication (FPF) is a polymer recycling method that has increased the use of 3D printing to make polymer filaments. Recycling of Polyethylene Terephthalate (PET) and Polypropylene (PP), the most common plastic wastes, was successful with no identifiable defects and negative effects on the mechanical properties of the reprocessed filaments.	The study affirms the reliability of FPF in recycling plastics and making 3D printing feedstock. It therefore contributes to prior evidence that 3D printing can be relied upon in recycling plastics.
[21]	A case study with an experimental research design.	PET	The mechanical properties of recycled Polyethylene Terephthalate (PET) change following Fused Deposition Modelling (FDM). The hardness of recycled PET decreased by 6% while the shear and tensile strengths increased by 2.8% and 14.7%, respectively, in comparison to the virgin PET. The tensile strength of recycled PET also reached a high of 43.15 MPa at a 3% elongation, suggesting that it is an ideal 3D printing material.	The study demonstrated the viability of FDM as a method of recycling PET wastes for 3D printing filaments. The findings also affirm the mechanical reliability of 3D printed filaments from recycled PET.
[79]	A quantitative study with an experimental design.	PLA, ABS, Nylon, PET, and PP	3D printing supports rapid prototyping, which has led to increase in production of 3D printed plastics. There has been an alarming rise in 3D printed plastic wastes due to the rapid growth in the 3D printing technology. Thermosets used in 3D printing are considered perilous to the environment and thus, alternative biodegradable 3D printable resins are being sought.	The study reveals that 3D printing can be used to support rapid prototyping and therefore increase the use of filaments from recycled plastics. In essence, the study provides evidence about the ways by which 3D printing promotes sustainable plastic waste management.
[74]	A quantitative study with an experimental design.	PLA	Recycling of PLA using FDM 3D printing technique results in production of biodegradable products with improved tensile and impact strength to up to 25.66% and 32.16%, respectively. The technique also reduces the water absorption rate of the recycled PLA material by up to 89.96%, rendering it a reliable method for making sustainable polymer products.	The study revealed that PLA can be recycled by the FDM method to make filaments with improved mechanical properties in comparison to the virgin PLA feedstock.
[94]	A quantitative research with an experimental design.	PVC	PVC undergo degradation by photolysis when exposed to ultraviolet light. The long chain hydrocarbons break, rendering a material brittle. However, the use of UV stabilizers such as Schiff bases reduces the vulnerability of PVC materials to photolysis.	The study demonstrates the likely solution to the limitations to use of polymers. The evidence is reliable in demonstrating how 3D printed filaments from recycled PVC can be made resistant to UV light to increase their durability and reduce their wastes in the surrounding.
[44]	A quantitative study with an experimental design.	PLA	Recycled PLA is turned into 3D printed filaments through melt extrusion with compression molding to industrial standards. However the heating process reduces polymer chains and increases the rate of degradation of the recycled polymers. However, polymerization at about 130 degrees Celsius improves the strength of the materials, rendering them durable and less likely to generate lots of wastes as it is with conventional virgin PLA material.	The findings demonstrate that PLA wastes can be turned into 3D printing filaments. The findings also show the likely defects of using hot melt extrusion in recycling PLA to make 3D printing filaments.
[78]	A quantitative study with an experimental design.	PET	Polyethylene terephthalate (PET) is the primary material for making packing products. Recycled PET is used in making filaments for 3D printing. It is also used in making 3D printed product prototypes. The materials exhibit thermally driven shape memory behavior evidenced by their recovery of their straight shape following heating and after being bent. The resulting material also has a high glass transition point and low recrystallization temperature. 3D printed PET can be recycled up to three times, an indication that 3D printed PET wastes are less likely to accumulate in the surrounding or case significant alarm because they can have a longer lifespan given the likelihood of recycling them multiple times.	The study suggests that commonly used plastics can be effectively recycled to make 3D printing filaments. The findings affirm that PET plastic wastes can be managed through their use in production of 3D printing filaments.
[102]	A mixed methods study with an experimental research design.	PLA	Plastic wastes from production of personal protective equipment are molded into filaments that are used for making 3D printed parts. 3D wastes from Fused Filament Fabrication (FFF) are also recycled and used as feedstock for 3D printers.	The evidence demonstrates recycling of plastic wastes from production of protective equipment by 3D printing. The study therefore provides reliable evidence regarding the use of 3D printing as a plastic recycling method.
[71]	A quantitative study with an experimental research design.	PLA	PLA is recycled by melt blending and transformed into strands that are used in 3D printing. However, the recycled PLA had high melt flow due to reduced molecular weight. The challenge particularly limited FDM 3D printing because of high extrusion flow. Nonetheless, the use of a chain extender for the materials improved its strength by up to 88%.	The study confirms the use of 3D printing in recycling plastics. Cisneros-López et al. (2020) affirmed FDM and HME are reliable methods for recycling polymers for production of 3D printing feedstock. However, the heating or melting process involved reduces the molecular weight of the material, suggesting a high likelihood of defects in 3D printing filaments produced from plastics recycled by HME and FDM.
[88]	Experimental study with quantitative and qualitative analysis of properties of 3D printed polymers suitable for greenhouse.	HDPE and Nylon	3D printing is used in the production of smart solar and greenhouse covers. The solar greenhouse covers made by 3D printing converted solar energy to electric energy and also provided optimum conditions under which plants grew.	The study demonstrates the viability of 3D printing in making reliable materials for agricultural applications. In ascertains the usefulness of 3D printing in production of advanced agricultural materials.
[89]	A quantitative method with an experimental design.	PET and PP	Synthetic plastics such as PET and PP from agricultural waste are recycled by co-polymer blending and alkali or acid treatment for production of functional components. Bio-based polymers are also synthesized from agricultural waste including fruits and vegetables. The availability of adequate agricultural waste has rendered the method scalable because it is cost-effective and has proven reliable in production of biodegradable polymers as a replacement for synthetic polymers for agricultural applications.	The study illustrates the recycling of plastic wastes from agricultural activities. The process’s efficacy is demonstrated by the fact that the wastes can be turned into 3D printing filaments and used as feedstock.
[90]	A mixed-methods study with an experimental research design.	Nylon	3D printed Nano-composite polymers, for example, Nylon 12 (PA12/TiO_2_) have lower mechanical strength but good antibacterial characteristics.	The study demonstrates how 3D printing is used to recycle plastic wastes. It also demonstrates the expected material properties for recycled plastic, in this case, Nylon.
[85]	A quantitative study with an experimental research design and quantitative analysis of recycled PLA material properties.	PLA	Repeated 3D printing of PLA leads to deterioration of its mechanical properties. Thus, repeated recycling of 3D printed products is nearly impossible, suggesting a likelihood of accumulation of 3D printed polymer wastes in the surrounding.	The findings suggest a likelihood of limited recycling cycles for PLA. The results contribute to an understanding of the limitations of 3D printing as a method of recycling plastics.
[70]	A mixed-methods study with thematic and statistical analysis.	Ocean plastic wastes	Plastics retrieved from oceans were converted into filaments by twisted screw extrusion to make feedstock for 3D printing.	The findings provided in-depth insights into the process of recycling ocean plastics and turning them into 3D printing filaments.
[69]	A quantitative method with an experimental design.	Plastics from landfills.	Plastics from landfills are collected and turned into filaments through extrusion for use in 3D printing. The process begins with shredding plastics into flakes that are hot extruded to create filaments. However, there is a likelihood of variability in strength due to the heat treatment of the materials.	The study provides evidence on the viability of hot melt extrusion in recycling used polymers and production of 3D printing filaments.
[75]	A quantitative analysis of the properties of virgin and recycled materials for 3D printing.	Virgin and recycled polymers	Virgin materials have superior mechanical properties. On the other hand, 3D printed filaments deteriorate on exposure to ultraviolet light, rendering them mechanically weaker. However they are preferred for production of cheaper 3D printed products.	The findings show that 3D printing is used in recycling plastics. It also adds to evidence that recycled plastic can deteriorate faster, demonstrating a limitation to use of 3D printing in recycling polymers.
[95]	Quantitative analysis of the properties of greenhouse covering material.	PVC and LLDPE	3D printing as an additive manufacturing technique is used in improving the UV transmittance of greenhouse covers with slow insecticide release and improved anti-microbial properties. PVC and LLDPE are also reinforced and stabilized with fillers to make them weather resistant and more durable.	The study shows how 3D printing can be used to enhance the properties of recycled polymers for agricultural applications. The findings therefore demonstrate how 3D printing can be leveraged in recycling plastic wastes.
[91]	A mixed methods study with analysis of the properties of 3D printed agricultural plastics.	Agricultural plastics	3D printing is employed in the production of polymer-based shed nets due to their heat resistance and anti-oxidation. Recycled polymers from agricultural plastics are usually collected, cleaned, and turned into flakes for hot extrusion into filaments for 3D printing.	The results from the study show how 3D printing is used as a recycling method.
[68]	An experimental study with Taguchi and ANOVA	Virgin and recycled Polypropylene	Polypropylene is extruded to make filaments that are used in 3D printing. However, the filaments from recycled propylene have a rough surface and easily bend.	The evidence demonstrated the feasibility of hot melt extrusion in recycling polypropylene polymers for 3D printing.
[80]	A quantitative study with an experimental research design.	PET	Recycled PET has properties similar to virgin PET. Thus, it is a reliable substitute material in making filaments for 3D printing. The materials have similar tensile strength, an aspect that suggests that they can be used as a replacement for virgin PET.	The study shows how hot melt extrusion can be utilized in production of 3D printing filaments and likely defects that might result from producing 3D filaments from polypropylene wastes.
[81]	A quantitative study with mechanical strength assessment experiments.	PLA	3D printing with recycled PLA is a viable option because it has properties similar to virgin PLA. For instance, the short beam strength of recycled PLA was 106 MPa with an allowance for 9 Mpa. On the other hand, virgin PLA had a short bean strength of 119 MPa plus or minus an allowable strength of 6.6 Mpa. Upon second recycling, PLA had a short beam strength of 108 MPa and 75 MPa following the third recycling. Arguably, the strength might deteriorate with the frequency of recycling.	The results from the study show the viability of 3D printing in recycling plastics to make feedstock with properties similar to the virgin material.
[93]	A quantitative study based on cycle analysis of HDPE.	Polystyrene	The weight and surface morphology of polystyrene changes following irradiation with UV light. However, upon addition of Schiff bases, polystyrene stabilized against UV radiation.	The study outcomes demonstrates how polymers can be protected against photo degradation to increase their longevity and limit their disposal as wastes in the surrounding.
[65]	A quantitative method with an experimental design.	ABS and HDPE	ABS and HDPE are granulated and hot extruded at 230 degrees Celsius to make 3D printing filaments. An assessment of the filaments demonstrated that a combination of 90% of ABS and 10% of HDPE produces filaments with consistent 3D prints.	The results from the study show how 3D printing is used as a recycling method. The study also shows how the properties of recycled polymers can be enhanced for durability and low plastic waste generation.
[45]	A quantitative method with an experimental design.	PLA	3D printing filaments from recycled PLA have a 10.9% less tensile strength, a 6.8% higher shear strength, and a 2.4% lesser hardness in comparison to virgin PLA. The modulus of elasticity remained unchanged for both the virgin and recycled PLA, an indication that recycled PLA filaments can retain their initial strength.	The results from the study show the viability of 3D printing in recycling plastics to make feedstock with properties similar to the virgin material.
[67]	A quantitative research with mechanical and structural experiments for PP.	PP	Polypropylene polymer cannot be recycled multiple times because their mechanical properties deteriorate with every subsequent hot extrusion at temperatures of 200 Celsius.	The results demonstrated the feasibility of hot melt extrusion in recycling polypropylene polymers for 3D printing. Therefore, it adds to available evidence about 3D printing as a plastic recycling method.
[86]	A quantitative study with an experimental research design.	PP	The mechanical properties of Polypropylene (PP) decline following recycling and extruding them into filaments. The elastic modulus decrease by 20%, while the elastic modulus decreases by 12.9%.	The findings suggest the limitation of 3D printing of recycled polymer filaments. It contributes to the rigor and diversity of evidence in assessment of 3D printing as a technique for recycling plastics.
[64]	A quantitative study based on the analysis of the mechanical properties of PLA composite from recycled PLA plastics.	PLA	PLA loses 50 to 60% of its strength following third time recycling. PLA recycled from virgin materials yields a 22% decline in mechanical strength in comparison to the original virgin PLA polymer. Only 85% of the strength is retained following first-time recycling.	The findings demonstrate a likely limitation to recycling polymers by 3D printing. The research outcomes contribute to an understanding of some of the challenges that one expects in using 3D printing as a recycling method.
[82]	A quantitative study involving testing and analyzing the mechanical behavior of recycled PLA.	PLA	Polyamide (PLA) losses viscosity by 15% for every recycling cycle. The change in viscosity after 10 recycling cycles results in a 70% decline in ultimate strength, 41% decrease in yield stress, a 38% decrease in young’s modulus, and 69% drop in fatigue resistance. However, changes after the first to the third recycling cycle are negligible, an indication that recycled PA can retain its properties at initial stages.	The findings demonstrate a likely limitation to recycling polymers by 3D printing. The research outcomes contribute to an understanding of some of the challenges that one expects in using 3D printing as a recycling method.
[84]	A quantitative study with an experimental research design.	PP and PLA	3D printed PLA losses its mechanical strength following thermomechanical treatment. Degradation was also noticed in its rheological and dimensional properties.	The findings demonstrate a likely limitation to recycling polymers by 3D printing. The research outcomes contribute to an understanding of some of the challenges that one expects in using 3D printing as a recycling method.
[63]	A quantitative method with an experimental design.	PLA	The strength of PLA significantly reduced following recycling. However, adding more PLA material during recycling yielded strengths of 52.61 MPa, representing an increase from 44.20 MPa for the virgin material. Thus, additive manufacturing can help enhance the mechanical properties of recycled PLA.	The findings demonstrate a likely limitation to recycling polymers by 3D printing. The research outcomes contribute to an understanding of some of the challenges that one expects in using 3D printing as a recycling method.
[66]	Mechanical fabrication with quantitative analysis.	Polystyrene and polypropylene	Mixing polypropylene and polystyrene wastes by Fuse Filament Fabrication yields 3D printing filaments with more than 32 MPa mechanical strength at extrusion temperatures of about 230 Celsius.	The study provides evidence on the viability of hot melt extrusion in recycling used polymers and production of 3D printing filaments.
[101]	A quantitative survey with structured questionnaires	Maritime plastic wastes.	Companies undertaking maritime operations acknowledge collecting, grading, and melting plastic wastes from oceans and seas for use in making 3D printing filaments. The move has particularly promoted recycling of oceanic plastic wastes and reduced their overall impact on aquatic life.	The study findings affirms that 3D printing can be used in recycling maritime plastic wastes.
